# Delta neutrophil index is an independent predictor of mortality in septic acute kidney injury patients treated with continuous renal replacement therapy

**DOI:** 10.1186/s12882-017-0507-8

**Published:** 2017-03-20

**Authors:** In Mi Han, Chang-Yun Yoon, Dong Ho Shin, Youn Kyung Kee, Seung Gyu Han, Young Eun Kwon, Kyoung Sook Park, Mi Jung Lee, Hyung Jung Oh, Jung Tak Park, Seung Hyeok Han, Shin-Wook Kang, Tae-Hyun Yoo

**Affiliations:** 1 Department of Internal Medicine, College of Medicine, Institute of Kidney Disease Research, Yonsei University, 50-1 Yonsei-ro, Seodaemun-gu, Seoul, 03722 Republic of Korea; 2 Department of Internal Medicine, Kangdong Sacred Heart Hospital, College of Medicine, Hallym University, Seoul, Republic of Korea; 3 Division of Nephrology, Department of Internal Medicine, Myongji Hospital, Seonam University College of Medicine, Goyang-si, Gyeonggi-do Republic of Korea; 4 Department of Internal Medicine, CHA Bundang Medical Center, CHA University, Seongnam-si, Republic of Korea; 5 Ewha Institute of Convergence Medicine, Ewha Womans University Mokdong Hospital, Seoul, Republic of Korea; 6 Severance Biomedical Science Institute, Brain Korea 21 PLUS, Yonsei University, Seoul, Republic of Korea

**Keywords:** Delta neutrophil index, Septic acute kidney injury, Continuous renal replacement therapy, Mortality

## Abstract

**Background:**

Delta neutrophil index (DNI), representing an elevated fraction of circulating immature granulocytes in acute infection, has been reported as a useful marker for predicting mortality in patients with sepsis. The aim of this study was to evaluate the prognostic value of DNI in predicting mortality in septic acute kidney injury (S-AKI) patients treated with continuous renal replacement therapy (CRRT).

**Method:**

This is a retrospective analysis of consecutively CRRT treated patients. We enrolled 286 S-AKI patients who underwent CRRT and divided them into three groups based on the tertiles of DNI at CRRT initiation (high, DNI > 12.0%; intermediate, 3.6–12.0%; low, < 3.6%). Patient survival was estimated with the Kaplan-Meier method and Cox proportional hazards models to determine the effect of DNI on the mortality of S-AKI patients.

**Results:**

Patients in the highest tertile of DNI showed higher Acute Physiology and Chronic Health Evaluation II score (highest tertile, 27.9 ± 7.0; lowest tertile, 24.6 ± 8.3; *P* = 0.003) and Sequential Organ Failure Assessment score (highest tertile, 14.1 ± 3.0; lowest tertile, 12.1 ± 4.0; *P* = 0.001). The 28-day mortality rate was significantly higher in the highest tertile group than in the lower two tertile groups (*P* < 0.001). In the multiple Cox proportional hazard model, DNI was an independent predictor for mortality after adjusting multiple confounding factors (hazard ratio, 1.010; 95% confidence interval, 1.001–1.019; *P* = 0.036).

**Conclusion:**

This study suggests that DNI is independently associated with mortality of S-AKI patients on CRRT.

## Background

Acute kidney injury (AKI) is a common and serious complication in critically ill patients [1, 2]. Septic AKI (S-AKI) accounts for close to 50% of all cases of AKI in the intensive care unit (ICU), and affects between 15 and 20% of patients in the ICU [3]. Continuous renal replacement therapy (CRRT) is an established treatment modality in critically ill patients with AKI in the ICU [4]. CRRT has several advantages in regard with the hemodynamic stability in patients with sepsis compared to intermittent renal replacement therapy including traditional dialysis and ultrafiltration therapy [5, 6].

In spite of potential advantages of CRRT in the management of S-AKI, the mortality rate in this patient group remains extremely high [7, 8]. To identify the predictors of mortality rate in S-AKI patients on CRRT treatment, several observational studies have been described [9, 10]. Previous studies focused on not only variable clinical factors but also sepsis or systemic inflammatory response syndrome (SIRS) related inflammatory mediators. Circulating pro- and anti-inflammatory cytokines, such as tumor necrosis factor (TNF)-α, interleukin (IL)-6, and IL-8, play an important role in the pathogenesis and progression of S-AKI and have been introduced as potential biomarkers of S-AKI. C-reactive protein (CRP) and procalcitonin were used to help predict mortality risk in patients with sepsis [11–13]. However, these biomarkers have not been found to be easily applicable due to limitations of timeliness and cost-effectiveness in critically ill S-AKI patients [12, 14].

Delta neutrophil index (DNI), calculated by subtracting the fraction of mature polymorphonuclear leukocytes from myeloperoxidase (MPO) reactive cells, represents proportion of circulating immature granulocytes (IGs). DNI is provided by an automatic hematologic analyzer ADVIA2120 (Siemens Healthcare Diagnostics, Forchheim, Germany) using MPO and nuclear lobularity channels [15]. A previous study demonstrated that, compared with white blood cells (WBCs) or CRP levels, DNI is a more useful marker for predicting mortality in patients with sepsis [16]. DNI has several advantages: it is simple, automatically reported, and rapidly recognized. However, little is known about the prognostic role of DNI in S-AKI patients, especially those treated with CRRT. Therefore, in this study, we explored whether high DNI is associated with high mortality rates in S-AKI patients receiving CRRT treatment at a single ICU center in Republic of Korea.

## Methods

### Study subjects

**Fig. 1:**
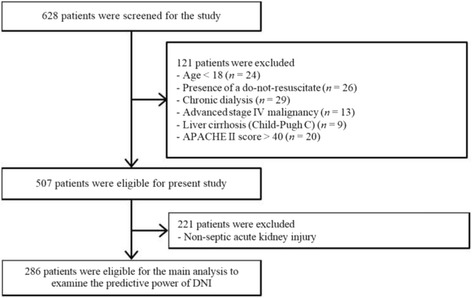
Flow diagram of the study. Abbreviations: APACHE, Acute Physiology and Chronic Health Evaluation; DNI, delta neutrophil index

All data from patients were retrieved from CRRT Database at Severance Hospital, Yonsei University Health System (YUHS) in Seoul, Republic of Korea. YUHS operates a specialized CRRT team (SCT), which includes physicians and nurses who are especially trained and educated in performing CRRT. Related details have been previously described [8]. Through a retrospective review of the consecutively registered CRRT Database, 628 patients who started CRRT from August 2011 to September 2013 were considered eligible for the present study. We excluded 121 patients who were younger than 18 years and/or the presence of a do-not-resuscitate (DNR) order. Because DNI values do not adequately work in immunocompromised individuals [17], the subject seems like has the components of immune suppression such as individuals those who have previously experienced chronic dialysis, or diagnosed with advanced stage IV malignancies, liver cirrhosis (Child-Pugh C), or higher than 40 points with Acute Physiology and Chronic Health Evaluation (APACHE) II score at enrollment were also excluded. The survival analysis according to the DNI groups was only performed with S-AKI populations (*n* = 286, Fig. 1).

To define the sepsis, we followed the consensus conference criteria from the American College of Chest Physicians/Society of Critical Care Medicine [18]. The individual those who had clinical findings supposed infection and simultaneously had 2 consecutive factors agree with SIRS, they diagnosed as the presence of sepsis. In SIRS, at least 2 of the following criteria were required: 1) core temperature > 38 °C or < 36 °C, 2) heart rate > 90 beats per minute, 3) respiratory rate > 20 breaths per minute, 4) arterial partial pressure of CO_2_ < 32 mmHg, 5) peripheral leukocyte count > 12,000/mm^3^ or < 4,000/mm^3^ [19]. To define SIRS, we used the results of vital signs and biochemical tests that were measured just before beginning of CRRT.

We included AKI patients with injury or failure stage of Risk, Injury, Failure, Loss of kidney function, and End-stage kidney disease (RIFLE) criteria at the time of CRRT initiation [20]. S-AKI was defined as SIRS combined with an infectious episode and renal dysfunction.

### Data collection

Depending on the protocol performed by SCT, all patients receiving CRRT were recorded with demographic and biochemical data including DNI at the beginning of CRRT, and these information were collected retrospectively for present analyses. Actually, blood samples comprising resource for DNI calculation was retrieved at the time immediately before the initiation of CRRT. Data of complete blood cell (CBC) counts were collected from an automated hematology analyzer (ADVIA 2120). DNI was calculated using the following formula: DNI (%) = (the leukocyte subfraction assayed in the MPO channel by cytochemical reaction) − (leukocyte subfraction counted in the nuclear lobularity channel by reflected light beam) [15]. Mean arterial pressure (MAP) was defined as diastolic pressure plus a third of the pulse pressure, and recorded at the time of initiating CRRT. For the assessment of disease severity, Sequential Organ Failure Assessment (SOFA) score, APACHE II score, and Charlson Comorbidity Index (CCI) were evaluated at the start of CRRT treatment. Comorbidities were defined by diagnosis codes based on the *International Statistical Classification of Diseases and Related Health Problems*, 10^th^ revision. Subjects who were taking antihypertensive or antidiabetic medications were also considered hypertensive or having diabetes, respectively. The following biochemical laboratory test result data were collected: hemoglobin, WBCs, platelet, blood urea nitrogen, serum creatinine, total cholesterol, serum albumin, high sensitivity CRP (hs-CRP), total bilirubin, prothrombin time (PT), and activated partial thromboplastin time (aPTT). The estimated glomerular filtration rate (eGFR) was calculated by using the chronic kidney disease epidemiology collaboration equation [21], and hs-CRP levels were determined by a latex-enhanced immunonephelometric method using a BNII analyzer (Dade Behring, Newark, DE, USA).

### ICU setting and CRRT protocol

The investigation site was a self-contained, 99-bed medical and surgical ICU in a 2076-bed teaching hospital in Seoul, Republic of Korea, equipped with 15 CRRT machines. CRRT initiation criteria include medically intractable and persistent electrolyte imbalance and/or metabolic acidosis, as well as decreased urine volume with overhydrated status and/or progressive azotemia. Hemodynamic instability was also considered an important factor in decision-making. However, the final decision to start CRRT was based on comprehensive judgment of various indications. Specifically, decisions to determine CRRT settings, which included target removal, blood flow, dialysate and replacement fluid rates, and the use of anticoagulant, were finalized by the nephrologist after close consultations and discussions between nephrologists, physicians, and ICU practitioners. Vascular accesses for CRRT have been selected among via the femoral, internal, or subclavian veins according to their availability and combined comorbidities. In most patients, continuous venovenous hemodiafiltration (CVVHDF) was performed using the PRISMA platform (Gambro, Hechingen, Germany). CRRT was initiated with a blood flow rate of 100 mL/min, which was gradually increased to 150 mL/min. The ultrafiltration dose was targeted to 40 mL/kg/h, and Hemosol (Gambro) was replaced using a predilution method. In addition, CRRT circuits were exchanged regularly every 48 h or when the blood pump was stopped. The patients were weighted every morning using an in-bed scale, and the ultratfiltration dose was adjusted [22].

### Statistical analysis

Statistical analysis was performed using IBM SPSS software for Windows version 23.0 (IBM Corporation, Armonk, NY, USA). Continuous variables were presented as means as standard deviation, and categorical variables as numbers and percentages. Patients were divided into three groups based on tertiles of DNI values at CRRT initiation: high, intermediate, and low DNI groups. The Shapiro-Wilk test was used to analyze the normality of the distribution of parameters. Baseline characteristics according the trichotomized DNI groups were compared using the one-way analysis of variance (ANOVA) for continuous variables and chi-square test for categorical variables. Nonparametric variables were expressed as median and interquartile range and compared using the Kruskal-Wallis test. To compare differences of continuous parameters between survivors and non-survivors, Student’s *t*-test or the Mann-Whitney *U*-test were used for parametric or non-parametric variables, respectively. Pearson correlation coefficients were used to assess the relationship between DNI and variable selected clinical parameters. In the present study, we evaluated 28-day all-cause mortality as an end point. Survival curves were generated by the Kaplan-Meier method, and between-group survival was compared by the log-rank test. We conducted receiver operating characteristic (ROC) analysis to compare the predictive accuracy of DNI and SOFA scores, and area under the curve (AUC) was calculated. In addition, we graded by DNI on a scale of 1–3 (DNI < 3.6%, 1; 3.6% ≤ DNI ≤ 12.0%, 2; DNI > 12.0%, 3) and added to the baseline SOFA score. The independent prognostic values of clinical parameters for the study outcome were analyzed by multiple Cox regression analysis. Hazard ratios (HRs) and 95% confidence intervals (CIs) were calculated with the use of the estimated regression coefficients and standard errors in the Cox regression analysis. The independent association of DNI levels for the 28-day all-cause mortality was confirmed by multiple logistic regression analysis. All probabilities were two-tailed and the level of significance was set at 0.05.

## Results

### Population characteristics

The demographic and biochemical characteristics of the study population with S-AKI are shown in Table 1. At the time of ICU admission, 129 (45.1%), 82 (28.7%), and 75 (26.2%) patients were classified as RIFLE-R, −I, and -F, respectively. Meanwhile, 177 (61.9%) patients were classified as RIFLE-I, and the remaining 109 (38.1%) patients were classified as RIFLE-F at the time of CRRT. DNI values ranged from 0 to 73.40%, with a median of 6.10%. When the patients were divided into three groups based on the tertiles of DNI level at CRRT initiation (high, DNI > 12.0%; intermediate, 3.6–12.0%; low, < 3.6%), patients with the highest tertile of DNI had higher APACHE II scores (27.9 ± 7.0 vs. 24.6 ± 8.3, *P* = 0.003) and SOFA scores (14.1 ± 3.0 vs. 12.1 ± 4.0, *P* = 0.001) compared to the lowest tertile group. Patients with the highest tertile of DNI had lower MAP, WBCs, platelet, and total cholesterol levels. In addition, they had more prolonged PT and aPTT. However, there were no significant differences in age, gender, CCI score, hemoglobin, eGFR, and hs-CRP among three groups.

**Table 1:** Baseline characteristics of patients according to delta neutrophil index

Variables	Total (*n* = 286)	1^st^ tertile (*n* = 94, < 3.6%)	2^nd^ tertile (*n* = 96, 3.6–12.0%)	3^rd^ tertile (*n* = 96, > 12.0%)	*P* value
Age (year)	61.0 ± 14.7	60.6 ± 15.5	62.2 ± 14.8	60.2 ± 13.8	0.626
Male (%)	180 (63.2)	58 (62.4)	57 (59.4)	65 (67.7)	0.480
MAP (mmHg)	75.9 ± 13.7	78.9 ± 15.7	75.7 ± 12.1	73.3 ± 12.7	0.016
APACHE II score	26.8 ± 7.6	24.6 ± 8.3	28.0 ± 7.2	27.9 ± 7.0	0.003
SOFA score	13.1 ± 3.7	12.1 ± 4.0	13.1 ± 3.8	14.1 ± 3.0	0.001
Charlson comorbidity score	13.1 ± 2.1	13.2 ± 2.2	13.0 ± 2.2	12.9 ± 1.8	0.611
RIFLE criteria (%)^*a*^					
Risk	129 (45.1)	42 (44.7)	45 (46.9)	42 (43.8)	0.666
Injury	82 (28.7)	25 (26.6)	31 (32.3)	26 (27.1)	
Failure	75 (26.2)	27 (28.7)	20 (20.8)	28 (29.2)	
Comorbid diseases (%)					
Hypertension	111 (38.8)	40 (42.6)	43 (44.8)	28 (29.2)	0.056
Diabetes mellitus	98 (34.3)	42 (44.7)	32 (33.3)	24 (25.0)	0.016
Biochemical data					
DNI (%)	6.10 (2.73–19.78)	1.35 (0–2.71)	6.00 (4.40–8.40)	27.9 (19.18–43.60)	<0.001^*b*^
Hemoglobin (g/dL)	9.6 ± 5.3	9.3 ± 2.2	9.4 ± 2.1	10.3 ± 8.6	0.349
WBC (10^3^/mm^3^)	11.6 (5.7–18.1)	13.6 (7.0–17.4)	13.9 (9.5–20.6)	7.5 (3.3–17.5)	0.002^*b*^
Platelet (10^3^/ mm^3^)	86 (51–163)	112 (62–207)	100 (61–185)	57 (41–94)	<0.001^*b*^
eGFR (mL/min/1.73 m^2^)	30.9 ± 17.0	27.9 ± 16.0	32.3 ± 17.2	32.5 ± 17.6	0.114
Cholesterol (mg/dL)	93.2 ± 47.8	104.7 ± 51.4	94.3 ± 44.8	81.4 ± 44.7	0.008
Albumin (g/dL)	2.7 ± 0.6	2.8 ± 0.6	2.7 ± 0.7	2.6 ± 0.6	0.088
hs-CRP (mg/L)	104.6 (34.3–199.8)	71.4 (19.7–160.1)	114.5 (44.0–196.8)	128.3 (35.6–279.9)	0.126^*b*^
Total bilirubin (mg/dL)	1.6 (0.7–3.6)	1.6 (0.7–4.7)	1.4 (0.6–3.6)	1.9 (0.7–3.4)	0.523^*b*^
PT (INR)	1.6 ± 0.7	1.6 ± 0.6	1.5 ± 0.6	1.8 ± 0.8	0.003
aPTT (sec)	48.9 ± 28.0	42.0 ± 16.2	44.1 ± 22.7	59.8 ± 36.9	<0.001

*Abbreviations*: *MAP* mean arterial pressure, *APACHE* Acute Physiology and Chronic Health Evaluation, *SOFA* Sequential Organ Failure Assessment, *DM* diabetes mellitus, *DNI* delta neutrophil index, *WBC* white blood cells, *Cr* creatinine, *eGFR* estimated glomerular filtration rate, *hs*-*CRP* high sensitivity C-reactive protein, *PT* prothrombin time, *aPTT* activated partial thromboplastin time, *ICU* intensive care unit

^*a*^At ICU hospitalization; At the time of CRRT, 177 (61.9%) patients were classified as RIFLE-I, and the remaining 109 (38.1%) patients were classified as RIFEL-F; ^*b*^Kruskal-Wallis test

When the study population divided into two groups according to their mortality events, non-survivor group showed significantly higher APACHE II (28.1 ± 7.3 vs. 24.0 ± 7.5, *P* < 0.001) and SOFA (14.2 ± 3.1 vs. 10.8 ± 3.7, *P* < 0.001) score, higher DNI [8.35 (3.23–24.73) vs. 3.95 (2.35–9.10) %, *P* < 0.001], PT (1.7 ± 0.8 vs. 1.5 ± 0.6 INR, *P* = 0.007), and aPTT (53.1 ± 31.9 vs. 40.4 ± 14.6 s, *P* < 0.001) levels compared with survivor group, while MAP (73.1 ± 12.8 vs. 81.7 ± 13.9 mmHg, *P* < 0.001), history of hypertension [65 (33.9%) vs. 46 (48.9%), *P* = 0.014] and diabetes [58 (30.2%) vs. 40 (42.6%), *P* = 0.039], WBCs [10.6 (4.0–17.5) vs. 13.9 (8.9–20.3) 10^3^/mm^3^, *P* = 0.009], platelet counts [80 (44–129) vs. 114 (57–204) 10^3^/mm^3^, *P* = 0.002], and serum albumin concentrations (2.6 ± 0.6 vs. 2.8 ± 0.7 g/dL, *P* = 0.013) were significantly lower in non-survivor group (Table 2).

**Table 2:** Baseline characteristics of patients according to the 28-day mortality event

Variables	Survivor (*n* = 94, 32.9%)	Non-survivor (*n* = 192, 67.1%)	*P* value
Age (year)	59.6 ± 14.9	61.7 ± 14.6	0.257
Male (%)	61 (65.6)	119 (62.0)	0.553
MAP (mmHg)	81.7 ± 13.9	73.1 ± 12.8	<0.001
APACHE II score	24.0 ± 7.5	28.1 ± 7.3	<0.001
SOFA score	10.8 ± 3.7	14.2 ± 3.1	<0.001
Charlson comorbidity score	12.9 ± 2.3	13.1 ± 2.0	0.347
RIFLE criteria (%)^*a*^			
Risk	48 (51.1)	81 (42.2)	0.345
Injury	23 (24.5)	59 (30.7)	
Failure	23 (24.5)	52 (27.1)	
Comorbid diseases (%)			
Hypertension	46 (48.9)	65 (33.9)	0.014
Diabetes mellitus	40 (42.6)	58 (30.2)	0.039
Biochemical data			
DNI (%)	3.95 (2.35–9.10)	8.35 (3.23–24.73)	<0.001^*b*^
Hemoglobin (g/dL)	10.5 ± 8.7	9.2 ± 2.2	0.063
WBC (10^3^/mm^3^)	13.9 (8.9–20.3)	10.6 (4.0–17.5)	0.009^*b*^
Platelet (10^3^/ mm^3^)	114 (57–204)	80 (44–129)	0.002^*b*^
eGFR (mL/min/1.73 m^2^)	76.7 ± 32.5	80.4 ± 29.2	0.404
Cholesterol (mg/dL)	98.1 ± 50.5	90.9 ± 46.5	0.271
Albumin (g/dL)	2.8 ± 0.7	2.6 ± 0.6	0.013
hs-CRP (mg/L)	106.0 (49.7–205.5)	102.6 (26.7–198.0)	0.581^*b*^
Total bilirubin (mg/dL)	1.4 (0.6–3.0)	1.9 (0.7–4.5)	0.101^*b*^
PT (INR)	1.5 ± 0.6	1.7 ± 0.8	0.007
aPTT (sec)	40.4 ± 14.6	53.1 ± 31.9	<0.001

*Abbreviations*: *MAP* mean arterial pressure, *APACHE* Acute Physiology and Chronic Health Evaluation, *SOFA* Sequential Organ Failure Assessment, *DM* diabetes mellitus, *DNI* delta neutrophil index, *WBC* white blood cells, *Cr* creatinine, *eGFR* estimated glomerular filtration rate, *hs*-*CRP* high sensitivity C-reactive protein, *PT* prothrombin time, *aPTT* activated partial thromboplastin time, *ICU* intensive care unit

^*a*^At ICU hospitalization; ^*b*^Mann-Whitney *U*-test

### Associations between DNI values and other parameters

There were significant negative correlations between baseline DNI values and MAP (*r* = −0.159, *P* = 0.007), platelets (*r* = −0.219, *P* < 0.001), and albumin (*r* = −0.150, *P* = 0.012). In contrast, there were positive correlations between baseline DNI values and APACHE II score (*r* = 0.119, *P* = 0.045), SOFA score (*r* = 0.166, *P* = 0.005), PT (*r* = 0.160, *P* = 0.008), and aPTT (*r* = 0.268, *P* < 0.001). There was no correlation in DNI levels with WBC, eGFR, and hs-CRP (Table 3).

**Table 3:** Correlation between baseline delta neutrophil index and selected clinical parameters

Variables	Delta neutrophil index (%)	
	γ	*P* value
Mean arterial pressure (mmHg)	−0.159	0.007
APACHE II score	0.119	0.045
SOFA score	0.166	0.005
WBC (10^3^/mm^3^)	0.030	0.609
Platelet (10^3^/mm^3^)	−0.219	<0.001
eGFR (mL/min/1.73 m^2^)	0.022	0.716
Albumin (g/dL)	−0.150	0.012
hs-CRP (mg/L)	0.135	0.073
PT (INR)	0.160	0.008
aPTT (sec)	0.268	<0.001

*Abbreviations*: APACHE, Acute Physiology and Chronic Health Evaluation; SOFA, Sequential Organ Failure Assessment; WBC, white blood cells; eGFR, estimated glomerular filtration rate; hs-CRP, high sensitivity C-reactive protein; PT, prothrombin time; aPTT, activated partial thromboplastin time

### Risk analysis for all-cause mortality

**Fig. 2:**
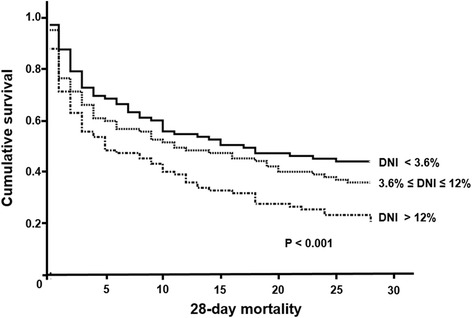
Kaplan-Meier plots for 28-day mortality-free survival rates. The comparison were conducted with trichotomized DNI groups. Abbreviations: DNI, delta neutrophil index

During the study period, 192 (67.1%) patients died. Twenty-eight-day mortality rate was significantly higher in the highest DNI group compared with intermediate and lowest DNI groups (80.2 vs. 64.6 vs. 56.4%, *P* < 0.001, Fig. 2). In Cox regression analysis in which DNI levels were treated as a continuous variable, DNI level was significantly associated with 28-day mortality (per 1% increase of DNI; HR 1.014; 95% CI, 1.007–1.022; *P* < 0.001) (Table 4, Model 1). After adjustment for age, gender, MAP, and SOFA score were included in a multivariate model, DNI remained an independent predictor of 28-day mortality (HR 1.011; 95% CI, 1.003–1.019; *P* = 0.010) (Table 4, Model 3). In addition, the significance was remained even more adjustment for platelet, albumin, PT, and aPTT (HR 1.010; 95% CI, 1.001–1.019; *P* = 0.036) (Table 4, Model 4). Meanwhile, we did not find any significant differences according to DNI levels in patients with non-S-AKI (data not shown, Model 4, *P* = 0.638).

**Table 4:** Association of delta neutrophil index for 28-day all-cause mortality in patients underwent CRRT

	Per 1% delta neutrophil index increase					
	Cox proportional hazard regression analyses			Logistic regression analyses		
	Hazard ratio	95% CI	*P* value	Odds ratio	95% CI	*P* value
Model 1	1.014	1.007–1.022	<0.001	1.034	1.013–1.055	0.001
Model 2	1.012	1.004–1.020	0.003	1.028	1.007–1.050	0.008
Model 3	1.011	1.003–1.019	0.010	1.024	1.003–1.046	0.027
Model 4	1.010	1.001–1.019	0.036	1.024	1.002–1.046	0.031

Model 1, crude model

Model 2, adjusted for age, gender, and mean arterial pressure

Model 3, Model 2 + SOFA score

Model 4, Model 3 + platelet, albumin, PT, and aPTT

*Abbreviations*: *CRRT* continuous renal replacement therapy, *CI* confidence interval, *SOFA* Sequential Organ Failure Assessment, *PT* prothrombin time, *aPTT* activated partial thromboplastin time

**Fig. 3:**
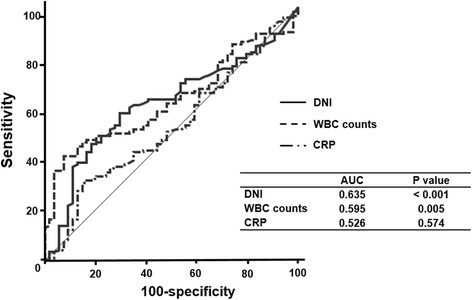
Receiver operating characteristics curve for the prediction of mortality event by DNI index. Abbreviations: DNI, delta neutrophil index; WBC, white blood cell; CRP, C-reactive protein; AUC, area under the curve

The relationship between the 28-day mortality rates and DNI values was confirmed by multiple logistic regression analyses with adjustments for multiple confounding factors (Table 4). In the fully adjusted model, increased DNI levels were still independently associated with the risk of 28-day mortality event in S-AKI patients [DNI, 1% increase, odds ratio (OR), 95% confidence interval (CI) = 1.024 (1.002–1.046), *P* = 0.031]. The ROC curves using variables (DNI value, hs-CRP, and WBC counts) are plotted in Fig. 3. The AUCs of DNI value and hs-CRP for 28-day all-cause mortality were 0.635 and 0.526, respectively (*P* < 0.001, Fig. 3).

## Discussion

This study demonstrated that DNI value is closely related to severity of disease in patients with S-AKI. In addition, baseline DNI level is independently associated with mortality in S-AKI patients treated with CRRT, even after adjusting for other established prognostic variables such as SOFA score.

DNI is the difference between the leukocyte differential assayed in the MPO channel and that measured in the nuclear lobularity channel, and was initially designed as a reliable and reproducible method to reflect IGs in circulating blood. The shift to the left of neutrophils, which reflects elevated IGs, has been characterized in sepsis and SIRS. Leukocyte count could be variable according to severity of sepsis in patients in the ICU. WBC count can increase in response to bacterial infection. Meanwhile, sepsis-associated leukopenia has been explained by impaired bone marrow production and peripheral overconsumption and/or destruction in response to disseminated intravascular coagulation (DIC). Several studies reported that DNI was closely related to sepsis severity [23], detection rate of blood cultures [24], DIC scores [15], and mortality in patients with suspected sepsis [16]. Moreover, another study showed that DNI may serve as a more useful diagnostic and prognostic marker than lactate for early diagnosis of disease severity in patients with septic shock [25]. More recently, leaving the usefulness of DNI on the field of critical medicine aside, DNI showed the association with several inflammatory status which could be overlooked by clinicians such as acute appendicitis, low-grade community-acquired pneumonia, or pyelonephritis in transplanted subjects [26–28]. There were no significant differences in WBC counts or neutrophil proportion among the groups categorized using DNI values. In addition, WBC counts alone did not predict patient outcomes. However, there were significant relationships between DNI and DIC-related parameters, including platelet count, PT, and aPTT. These findings added to the evidence that baseline DNI is a significant determinant of mortality in AKI patients requiring CRRT. In addition, DNI is routinely performed and automatically calculated without additional costs. DNI values can be rapidly recognized in the CBC report. Taken together, we surmised that DNI could be an early and potent prognostic indicator in patients with S-AKI.

Although DNI as a prognostic marker for sepsis might be comparable to other pro-inflammatory cytokines such as CRP and procalcitonin, DNI can change in conditions of ineffective leukocyte production. Since the production of IGs and DNI values could be suppressed in immunocompromised patients, DNI value alone could not discriminate between bacteremia and non-bacteremia in these patients. In addition, the production of IGs may be altered in neonates, pregnant women, and patients with other hematologic diseases or bone marrow alterations. Under these conditions, DNI should be interpreted with caution, and other biomarkers, such as CRP and procalcitonin, might be included to assess the severity of SIRS or sepsis.

Even though the present study measured IGs using the ADVIA automatic analyzer, several other hematology analyzers can obtain IG counts. The Cell-Dyn series analyzer counts the IG fraction using a 4-dimensional optical scanner and multi-parameter flow cytometry, and the Sysmex analyzer also enumerates IG counts using the difference of fluorescence between mature and immature granulocytes, similar to the ADVIA analyzer. Further comparative analyses among these techniques might be conducted to validate the clinical usefulness in sepsis patients including S-AKI patients.

Predictive scoring systems have been developed to measure the severity of disease and the prognosis of patients with sepsis in the ICU [29, 30]. However, these scoring systems have important limitations, such as inaccuracy according to the type of disease [31–33], lead time bias [34], and need for updating [35]. The accuracy of prediction of mortality with severity scoring indices alone is relatively poor. Therefore, other factors such as serologic makers related to sepsis must be considered together to predict the outcome in severe sepsis. A recent study demonstrated that pro-inflammatory mediators such as TNF-α, IL-1ß, and IL-6 are important clinical prognostic markers in patients with systemic sepsis. There is a strong correlation between serum concentration of pro-inflammatory mediators and mortality in septic patients [36]. In spite of this advantage, most of these mediators are not established for clinical decision-making due to their short half-life. In addition, the same goal could be achieved more easily and cheaply by the estimation of blood lactate level.

AKI has long been considered primarily as a hemodynamic condition characterized by a reduction of renal blood flow, induced by either cardiogenic or septic shock [37]. However, Bellomo et al. produced new and interesting data in animal models of S-AKI that undermined existing concepts. They observed that medullary and cortical renal blood flow were both maintained and even increased in septic shock, underscoring that S-AKI was a totally different physiological phenomenon than non-S-AKI [38]. Inflammation is now believed to play a major role in the pathophysiology of S-AKI [39]. In fact, it is known that TNF-α can induce AKI [40], and Benes et al. found an early increase in TNF-α in animals developing S-AKI [41]. In addition, recent work highlighted the fact that inflammatory apoptosis could play a more important role in sepsis and septic shock than pure necrosis [42]. Interestingly, Simmons et al. suggested that an increase in plasma pro-inflammatory cytokine levels could predict mortality in patients with AKI. However, unlike other sepsis studies which showed that pro-inflammatory cytokines like IL-1ß and TNF-α could predict mortality, this study did not confirm such a relationship. Instead, IL-6 and IL-8, which were often considered secondary in the inflammatory cascades involving IL-1ß or TNF-α, were proven to be significant predictors of mortality. The authors suggested that renal failure may in and of itself confer an altered cytokine profile even in the context of critical illness. Another potential explanation for this discrepancy may be the timing of cytokine determination in the overall course of illness [43]. Therefore, the extent to which inflammatory mediators affect the prognosis in patients with S-AKI remains uncertain.

This study has several limitations. First, we did not evaluate follow-up data for DNI levels and thus could not account for possible variation over time. Since DNI levels can change according to the response to optimal treatment such as antibiotics, repeated measurement of DNI can serve as a helpful predictor of prognosis in patients with sepsis or SIRS. Further study to clarify the relationship of changes in DNI levels over time with prognosis might be valuable. Second, there was no investigation of pro-inflammatory cytokines related to sepsis in patients with S-AKI. Pro-inflammatory cytokines such as TNF-α, IL-1ß, and IL-6 play a more important role in the pathogenesis of sepsis and septic shock. In addition, there is a strong correlation between serum concentration of pro-inflammatory mediators and mortality in septic patients. The investigation of pro-inflammatory cytokines compared with DNI might be helpful in predicting outcomes in patients with S-AKI in the future. Third, DNI values have limitations for assessing bacteremia in immunocompromised individuals, thus there might be inadequate estimation in patients with severe immune suppression [17]. To mitigate these bias we initially excluded subjects those who considered immunocompromised. Lastly, in a group with high risk of death, clinical significance of the statistically significant relationship between DNI and mortality is limited. In this study, AUC of DNI was higher than that of WBC or CRP; however, its absolute value was still low. Future development of prediction models, including DNI through various approaches, will be helpful for such high-risk patients.

## Conclusions

In conclusion, the present study revealed that a greater increase in DNI levels is significantly associated with severity of disease and high mortality rates in S-AKI patients treated with CRRT. DNI is an early identifiable and reliable serologic maker to predict outcomes in patients with S-AKI requiring CRRT. Patients with high DNI levels should be cautiously monitored and treatment strategies should be appropriately adapted for their future needs.

## Abbreviations

AKI: Acute kidney injury
ANOVA: Analysis of variance
APACHE: Acute Physiology and Chronic Health Evaluation
aPTT: Activated partial thromboplastin time
AUC: Area under the curve
CBC: Complete blood cell
CCI: Charlson Comorbidity Index
CI: Confidence intervals
CRRT: Continuous renal replacement therapy
CVVHDF: Continuous venovenous hemodiafiltration
DIC: Disseminated intravascular coagulation
DNI: Delta neutrophil index
DNR: Do-not-resuscitate
eGFR: Estimated glomerular filtration rate
HRs: Hazard ratios
hs-CRP: High sensitivity C-reactive protein
ICU: Intensive care unit
IGs: Immature granulocytes
IL: Interleukin
MAP: Mean arterial pressure
MPO: Myeloperoxidase
PT: Prothrombin time
RIFLE: Risk, Injury, Failure, Loss of kidney function, and End-stage kidney disease
ROC: Receiver operating characteristic
S-AKI: Septic acute kidney injury
SCT: Specialized continuous renal replacement therapy team
SIRS: Systemic inflammatory response syndrome
SOFA: Sequential Organ Failure Assessment
TNF: Tumor necrosis factor
WBCs: White blood cells
